# A gradient-based optimization model for predicting decompression sickness risk

**DOI:** 10.3389/fphys.2026.1852640

**Published:** 2026-06-18

**Authors:** Sergio Rhein Schirato, Massimo Pieri, Riccardo Pelliccia, Alessandro Marroni, Costantino Balestra, José Guilherme Chaui-Berlinck

**Affiliations:** 1 Department of Physiology, Biosciences Institute, University of São Paulo, São Paulo, Brazil; 2 Divers Alert Network (DAN) Europe Research Division, Roseto degli Abruzzi, Italy

**Keywords:** decompression sickness, hyperbaric environment, optimizations algorithms, probabilistic models, SCUBA

## Abstract

Decompression sickness (DCS) is a low-incidence but potentially severe consequence of hyperbaric exposure. Probabilistic decompression models offer a framework to quantify this risk, yet their calibration is challenged by the scarcity of empirical outcome data. In this study, we propose a gradient-based optimization model to predict DCS probability, trained on 924 dive profiles from the US Navy Experimental Diving Unit XVal-He-9 tables, representing predefined DCS probabilities (2.3% and 4%), and optimized based on actual body tissues grouped in five compartments. The model achieved high predictive accuracy (MAE: 0.535%; RMSE: 0.694%) with consistent performance across training and test sets, indicating limited overfitting. Reduced accuracy was observed in intermediate depth ranges (100–130fsw or 30–39msw). Out-of-sample evaluation on 31 high-risk dives (three DCS cases) showed general agreement between predicted and observed incidence while suggesting a potential contribution of repetitive exposures not accounted for in the model. These results demonstrate that gradient-based optimization, trained based on existing probabilistic tables, seems to be capable of satisfactorily predicting decompression sickness risk for a given dive profile. Additionally, future studies can further adjust the loss function to account for individual or dive-related indicators, leading to a more individualized risk function.

## 1 Introduction

Decompression sickness (DCS) occurrence in self-contained underwater breathing apparatus (SCUBA) diving is a relatively rare but potentially life-threating event. The physiological pathways that lead to the event are still not completely understood, although a lot of progress has been made in recent years when not only the physics behind tissue saturation but also the physiological responses, in terms of production of pro-inflammatory factors and immune responses (Thom et al., 2014; Yang et al., 2015; Thom et al., 2018; Schirato et al., 2024), have been accounted for (Schirato et al., 2022).

The overall incidence of DCS is highly dependent on the decompression profile followed by a certain exposure to hyperbaric environments. Since the beginning of the 20th century, when The Prevention of Compressed-air Illness (Boycott et al., 1908) was published, deterministic models have been trying to produce different decompression strategies to reduce the risk of the development of decompression sickness. The outcomes, however, are dependent on the dive profile, and the variance in the DCS incidence varies greatly for the same deterministic model, according to the intensity of the exposure, in a way that the same algorithm can produce greatly different outcomes as the severity of the exposure varies (Theron et al., 2025; Blasselle et al., 2019).

In the past three decades, probabilistic models offered an alternative where the risk of a given exposure could be targeted in the process of building the decompression profile, in a manner that exposure was translated into a risk function (Thalmann et al., 1997) (Silvanius et al., 2023), based on tissue supersaturation, calculating within a confidence interval the probability of appearance of DCS symptoms. Over time, different strategies have been tested, assuming different gas absorption and elimination kinetics (Murphy et al., 2017; Murphy et al., 2018), or tried to estimate the severity of the symptoms in non-binary models (Howle et al., 2017) (Buzzacott et al., 2014). Differently than deterministic models, probability-based decompression models can offer a known variance in the DCS incidence across a variety of exposures. These models are built based on extensive empirical datasets of known profiles and DCS occurrence, fitted to a risk function through an optimization process.

Alternatively, different studies have tried to relate the occurrence of DCS symptoms to levels of compartment supersaturation, either at depth during ascent or at reaching the surface at the end of the dive (Cialoni et al., 2017), observing that higher levels of compartment supersaturation, when calculated in relation to Bühlmann’s maximum values (Bühlman, 1984), have a positive correlation with higher DCS incidence. These studies also relate higher incidences of DCS to individual factors, such as age, gender, and body composition, among others (Schirato et al., 2024). Differently than probabilistic models, where the objective is to estimate the risk of DCS in a population, the last are focused on individual behaviors or characteristics that would increase the probability of DCS incidence on an individual level. Therefore, the aim of these studies is to identify idiosyncratic features that would increase the probability of DCS incidence at an individual level.

In parallel with the probabilistic approach, there have been attempts to elaborate a reverse risk function for a given decompression profile from the observed probability of DCS (Thalmann et al., 1997) (Doolette et al., 2019; Survanshi, 1993; Murphy et al., 2017; Murphy et al., 2018). The main difference is that, now, the inputs for a certain decompression profile are the probability of DCS, the supersaturation threshold, and a relative relevance of a given theoretical compartment in the outcome of DCS. Then, the risk function obtains putative inert-gas dynamics (i.e., inert-gas half-time rate) for three theoretical compartments which can be employed to give estimated DCS probabilities in a new condition (e.g., depth and bottom time of a dive). Notably, the gas dynamics obtained by the approach just described are related to abstract, non-existing physiological body tissues, and the result is another probability of DCS.

The goal of the present study is to produce a probabilistic model for decompression risk prediction, such as that previously made by Thalmann et al. (1997) (Survanshi, 1993), among others (Doolette et al., 2019; Murphy et al., 2017; Murphy et al., 2018). However, in the present paper, instead of having the half-times calculation as part of the optimization process, risk functions were based on actual body tissues grouped in five compartments (Schirato et al., 2022) while assuming the half-times for helium and nitrogen to be the same (Doolette et al., 2005) The intercept and the angular coefficient of a linear latent risk score was added to the optimization in such a manner that different dive profiles can be compared in terms of expected risk of provoking DCS (Doolette et al., 2015). Additionally, it must be considered that XVal-He-9 tables are themselves the result of an optimization process, and as such, actual P(DCS), observed if the profiles were tested enough, might differ than the P(DCS) calculated using the XVal-He-9 algorithm.

## 2 Methods

### 2.1 Procedure overview

The present model was trained based on the XVal-He-9 tables published by the US Navy Experimental Diving Unit (Doolette et al., 2018), instead of relying on empirical data. The approach adopted was a gradient descent optimization process, where a set of 12 parameters was optimized to converge the calculated probability of DCS for a given dive profile to a pre-calculated probability given by the XVal-He-9 table. Based on the reasonable assumption that the XVal-He-9 calculated probabilities are accurate, although they are themselves the results of an optimization process, the utilization of this dataset produces an extremely robust model since the 924 profiles used in the training and validation processes correspond to the analysis of approximately 35,000 real dives (estimating a DCS incidence of 3% on average).

A dataset containing 924 dive profiles published by the Navy Experimental Diving Unit, calculated to produce P(DCS) of 2.3% and 4% (Doolette et al., 2018) throughout a range of depths and bottom times while using constant 1.3 PO_2_ (Hoyt et al., 2025), was used to calibrate the tissue sensitivity *G_i_* and supersaturation thresholds *Θ_i_* of a risk function as defined by Thalmann et al. (1997) Tissue inert−gas pressure dynamics are modeled using asymmetric kinetics: exponential uptake during on−gassing and transition to linear washout during off−gassing, when tissue supersaturation exceeds a compartment-specific crossover pressure (*P_xo_*), as developed by Thalmann and further refined by Howle (Thalmann et al., 1997; Howle et al., 2017); otherwise, it is exponential. The crossover pressure values used in this study were extracted from LE1 models (Thalmann et al., 1997) and converted from the original notation to ATA (2.42fsw equals 0.073 ATA).

The dataset was divided into parts of 616 and 308 profiles for training and testing, respectively, assuring equal participation of the two P(DCS) groups in the training. The code was designed to have 2,000 training cycles (epochs), subsequently applying the trained parameters to the test set.

Calculations were based on a five-tissue model, as previously published (Schirato et al., 2022). Body compartments that have approximately the same half-times were combined in a single compartment, creating a model of five compartments, as detailed in **Table 1**. Additionally, a descent time of 20 m per minute was added in the calculations, as well as an ascent time of 9 m per minute ascent velocity until the first decompression stop was reached.

**Table 1:** Compartment half-times.

Organs	Compartment	Half-time (min)
CNS, liver, kidneys	1	2.4
Heart, skin	2	9.5
Skeletal muscles	3	25.1
Gastrointestinal tract	4	74.3
Adipose tissues	5	307.5

Given the discrete nature of the datasets, where time is given in whole minutes, original on- and off-gassing differential equations were adjusted to the discrete, minute-based calculations. Exponential gas dynamics were calculated when *P_t_*,*_i_* ≤ *P_amb_*,*_t_* + *P_XO_* – *P_FVG_* or when *P_insp_* ≥ *P_t_*,*_i_* is described as:

(1) $$P_{t,i}\left(t+\Delta t\right)=P_{t,i}\left(t\right)\cdot e^{\left(-k_{i}\Delta t\right)}+P_{insp}\left(t\right)\cdot \left[1-e^{\left(-k_{i}\Delta t\right)}\right]$$

Linear dynamics, when *P_t_*,*_i_* > *P_amb_*,*_t_* + *P_XO_* – *P_FVG_* and *P_insp_*< *P_t_*,*_i_*, is given by:

(2) $$P_{t,i}\left(t+\Delta t\right)=P_{t,i}\left(t\right)+k_{i}\Delta t\cdot \left[P_{insp}\left(t\right)-P_{amb}\left(t\right)-P_{XO}-P_{FVG}\right]$$

When tissue pressure crosses below *P_amb_*(*t*) + *A_i_*, kinetics transition from linear back to exponential for the remainder of that interval.

For $K_{i}=\ln\left(2\right)T_{\frac{1}{2},i}^{-1}$ is the rate constant associated with tissue half−time *T*_1/2_.

The instantaneous relative supersaturation ratio *S_i_*,*_t_*(*t*) above the threshold *Θ_i_* is the core driver risk accumulation, as given by Equation 3.

(3) $$S_{i,j}\left(t\right)=max\left(P_{t,i}\left(t\right)-P_{amb}\left(t\right)-\Theta_{i},0\right)$$

(4) $$w_{j,i}\left(t\right)=\frac{S_{i,j}\left(t\right)}{P_{amb}}$$

The time-integrated overpressure for a tissue *i* is given by Equation 5:

(5) $$A_{j,i}=\int_{1}^{t}w_{i,j}\left(t\right)\;dt$$

The model’s exposure functional A*_j_*,*_i_* for profile *j* and compartment *i* is equivalent to the time integral of the supersaturation ratio, integrated over the profile duration *t*.

The total accumulated risk R_i_,*_j_*, given by Equation 6, is the sum of weighted compartment exposures and calculated using Equation 4 above or its simplified form, as shown below:

(6) $$\begin{array}{c} R_{1,j}=G_{i}\int_{0}^{t}\frac{P_{t,i}\left(t\right)-P_{amb}\left(t\right)-\Theta_{i}}{max\left(P_{amb}\left(t\right),0.5\right)}\;or,\;simply\\ R_{1,j}=\sum_{i=1}^{5}A_{j,i}\cdot G_{i} \end{array}$$

The model is constrained by as sigmoid function σ(*x*) so that the parameters represent a finite positive, as defined by Equations 7–9, guarantying that 0< *G_i_*< *G_max_* and 0< Θ_i_< Θ_max_, as per the equations below:

(7) $${\text{G}}_{\text{i}}={\text{G}}_{\max}\cdot \sigma \left({\text{g}}_{\text{i}}\right)$$

(8) $${\text{Θ}}_{\text{i}}={\text{Θ}}_{\max}\cdot \text{σ}\left({\text{θ}}_{\text{i}}\right)$$

(9) $$\sigma \left(x\right)=\frac{1}{1+e^{x}}$$

Additionally, β is constrained by *β* = *softplus*(*β*_1,_*_raw_*), where *softplux* (*x*) = ln(1 + *e^x^*), assuring that *β* > 0.

Probability of DCS.

A linear predictor *Z_j_* was calculated as a baseline linear score, as demonstrated by Equation 10, combining risk (*R*_1,_*_j_*) and exposure, in the format of:

(10) $$Z_{j}=\alpha +\beta R_{1,\;j}$$

The latent non-negative risk rate associated with dive profile *j*, representing the cumulative physiological hazard generated by inert-gas supersaturation exposure across tissues, is given by:

(11) $$R_{j}=e^{\left(clip\left(Z_{j},-15,5\right)\right)}$$

where *Z_j_* is clipped to assure numerical stability.

The base probability *P*(*DCS*)*_j_* for a profile *j* is given by:

(12) $${\hat{P}}_{j}=1-e^{-\left(R_{j}\right)}$$

where ${\hat{P}}_{j}$ was clipped to (10^-7^, 1-10^-7^) to ensure numerical stability to the loss function.

In this process, the linear predictor *Z_j_* is transformed into a non-negative event rate (Equation 11), which is then mapped to the predicted DCS probability (Equation 12), corresponding to the probability of at least one event in a Poisson process with rate *R_j_*. **Table 2** provides a conceptual summary of this linear adjustment.

**Table 2:** Symbols and their respective meaning.

*N*	Number of dive profiles
*j*	Profile index
*m*	Number of tissue compartments
*i*	Tissue compartment index
*t*	Time (min)
Δ*t*	Time step (min)
P_xo,i_	Crossover pressure, vector [0, 0.073, 0, 0, 0] (ATA)
P_t,i_(t)	Inert gas tissue pressure (ATA)
P_CO2_	0.07 ATA
P_FVG_	Fixed venous gas pressure at 0.19 (ATA)
P_insp_(t)	Inspired inert -gas pressure (ATA)
P_amβ_(t)	Ambient pressure (ATA)
T_1/2,i_	Tissue half -time (min)
k_i_	$\ln\left(2\right).{\text{T}}_{\frac{1}{2},\text{i}}^{1}{\left(\min\right)}^{-1}$
Θ_i_	Supersaturation threshold (ATA)
G_i_	Tissue sensitivity weight (min)^-1^
A_j,i_	Integrated supersaturation exposure (min)
R_1,j_	Cumulative exposure metric
S_max,j_	Maximum instantaneous normalized stress
S_j_	Log -transformed peak stress (diagnostic)
*α*	Intercept parameter
*β*	Linear coefficient
Z_j_	Linear predictor
R_j_	Non -negative latent risk rate
P_j_	Observed DCS probability
${\hat{P}}_{\text{j}}$	Predicted DCS probability
ℒ	Loss function

In the optimization process, the raw parameters (G*_i_*, Θ*_i_*, *α*, and *β*) had their initial values set to zero and were optimized using cross-entropy for Bernoulli (CE), as defined below, between ${\hat{P}}_{j}$ and *P_j_*, where ${\hat{P}}_{j}\in \left(0,1\right)$ and *P_j_* ∈ (0, 1), as given by Equation 13.

(13) $$CE\left(P_{j},{\hat{P}}_{j}\right)=-\left[P_{j}\log\left({\hat{P}}_{j}\right)+\left(1-P_{j}\right)\log\left(1-P_{j}\right)\log\left(1-{\hat{P}}_{j}\right)\right]$$

CE is adjusted for Kullback–Leibler divergence, which was used to correct for how much probability mass the model misallocates relative to the observed probabilities (King et al., 2001), ensuring that the fitted model preserves the correct risk distribution rather than merely matching average error, as defined by:

(14) $$D_{KL}\left(P_{j}||{\hat{P}}_{j}\right)=P_{j}\log\frac{P_{j}}{{\hat{P}}_{j}}+\left(1-P_{j}\right)\log\frac{1-P_{j}}{1-{\hat{P}}_{j}}$$

In other words, Kullback–Leibler divergence was used as a calibration metric to penalize deviations between predicted and empirically observed decompression risk probabilities, thereby encouraging the model to reproduce not only the average risk levels but also the overall probability structure of the reference datasets.

The loss function ℒ incorporated L2 regularization, as per Equation 15, to ensure that the penalty is always positive and that large values are penalized more strongly than the smaller ones. The loss function is given by:

(15) $$ℒ=\lambda_{ce}\frac{1}{N}\sum_{j=1}^{N}w_{j}\left[-P_{j}\log\left({\hat{P}}_{j}\right)-\left(1-P_{j}\right)\log\left(1-P_{j}\right)\log\left(1-{\hat{P}}_{j}\right)\right]+\lambda_{G}\sum_{i}G_{i}^{2}+\lambda_{\Theta }\sum_{i}\Theta_{i}^{2}+\lambda_{\beta }\beta^{2}$$

where,


$$\lambda_{ce}=1$$

$$\lambda_{G}=\;\lambda_{\text{Θ}}=\;10^{-3}$$

$$\lambda_{\beta }=\;10^{-2}$$

$$w_{j}=\;\{\frac{\frac{1}{N_{2.3\%}}}{\frac{1}{N_{4\%}}}$$

Gradients of the loss with respect to all raw parameters are computed by backpropagation via automatic differentiation, and parameters are updated using the Adam optimizer (Kingma and Ba, 2017), which applies bias-corrected adaptive moment estimates of first and second gradient statistics. All computations were performed in R using the Torch framework with CUDA acceleration.

### 2.2 Out-of-sample application

Once the model was trained, it was applied to a set of 31 real dives, which are similar in terms of overall exposure, that resulted in three cases of decompression sickness, with the objective of evaluating whether the results predicted by the model were within the boundaries of a small set of observations with a high likelihood of resulting in DCS. These dives were executed by scientific divers who gently allowed the authors to monitor and record their data.

## 3 Results

### 3.1 Training and testing results

**Figure 1:**
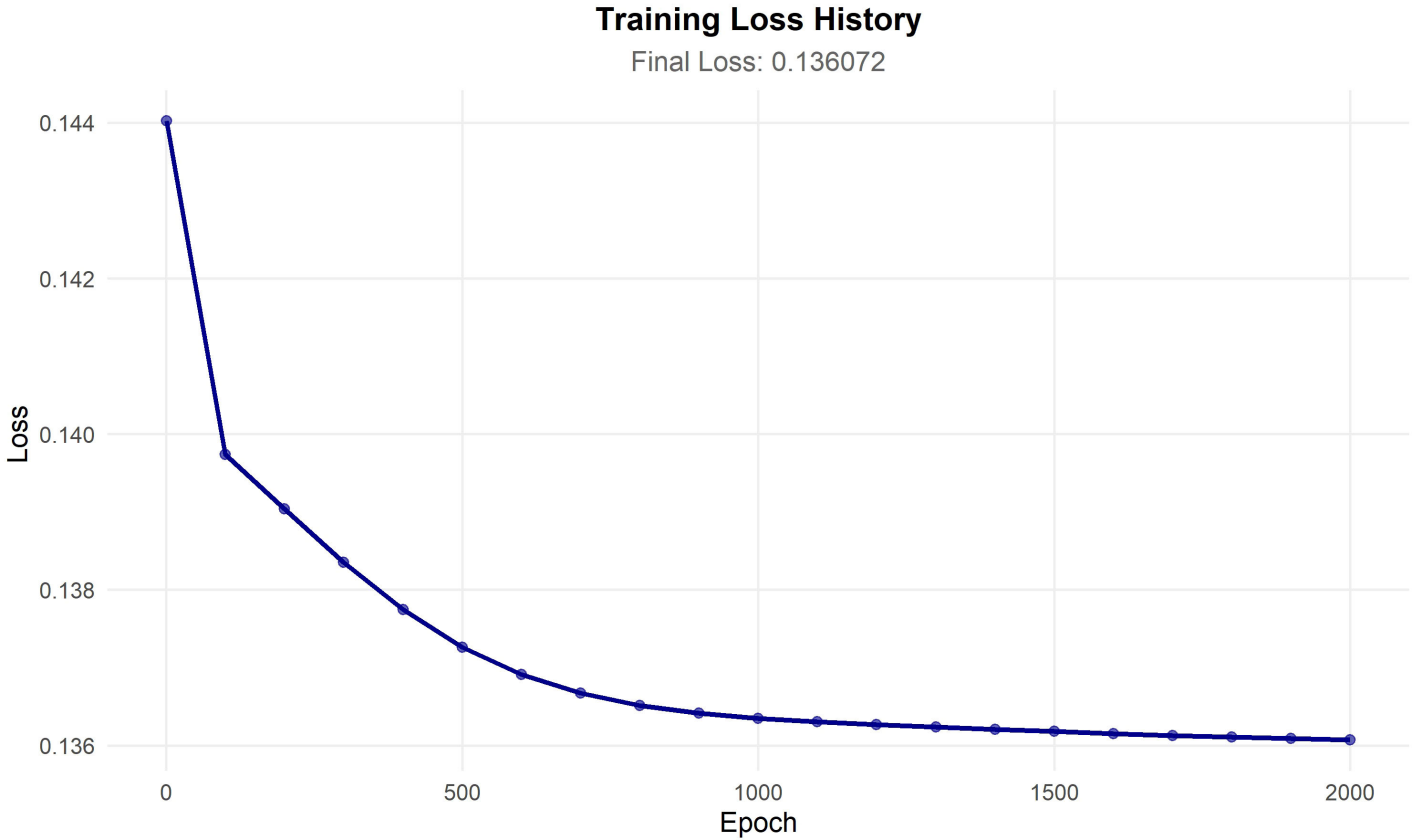
Total loss training trajectory logged every 100 epochs.

The evolution of the total loss, described as a weighted objective value of error and penalties, during the training process can be viewed in **Figure 1**.

Trained values for *θ_i_*, *G_i_*, *α*, and *β* are presented in **Table 3**, while the model’s accuracy is summarized in **Table 4**. The consistency of the observed errors, as measured using MAE and MSE, across the training and testing phases indicates that the gradient-descent optimization did not result in overfitting.

**Table 3:** Summary of the linear adjustment process.

Stage	Symbol	Domain	Meaning
Linear predictor	Z_j_	(-∞, ∞)	Calibrated exposure score
Latent rate	R_j_	[0, ∞)	Underlying DCS event
Probability	${\hat{P}}_{j}$	[0, 1]	Predicted DCS probability

**Table 4:** Trained parameters of the model.

Tissue sensitivity weight		Supersaturation threshold		Linear predictors	
G1	0.00323	Θ1	0.0657	α	-3.68531
G2	0.0326	Θ2	0.694	*β*	0.677837
G3	0.0188	Θ3	0.72		
G4	0.032508	Θ4	0.245		
G5	0.0235	Θ5	0.706		

**Figure 2:**
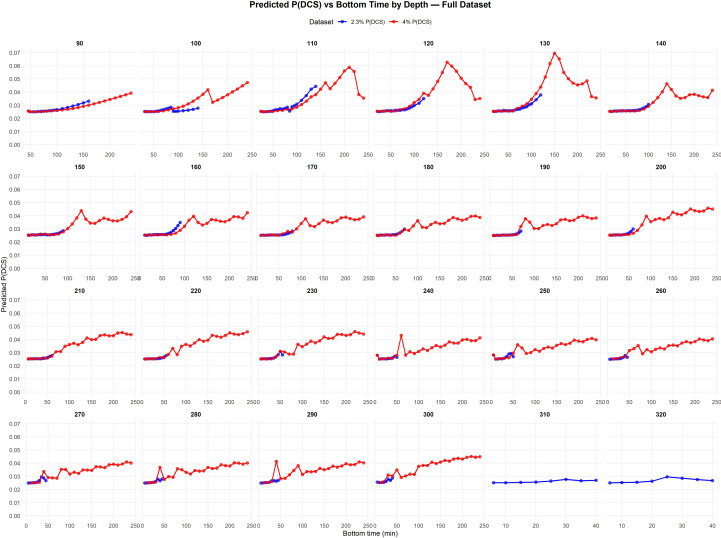
Predicted P(DCS) according to bottom time and depth for the dive profiles with 4% observed P(DCS). A lower accuracy is observed in the 100fsw (30msw) to 130fsw (39msw) profiles, in an exacerbation of the effect observed in the lower P(DCS) profiles.

An interesting outcome of the analysis was the reduced model accuracy for profiles in the depth range between 100fsw (30msw) and 130fsw (39msw), as can be viewed in **Figure 2**, where the model is overestimating P(DCS) when compared to the values contained in the XVal-He-9 tables used for training in some cases by a factor of 2×.

**Figure 3:**
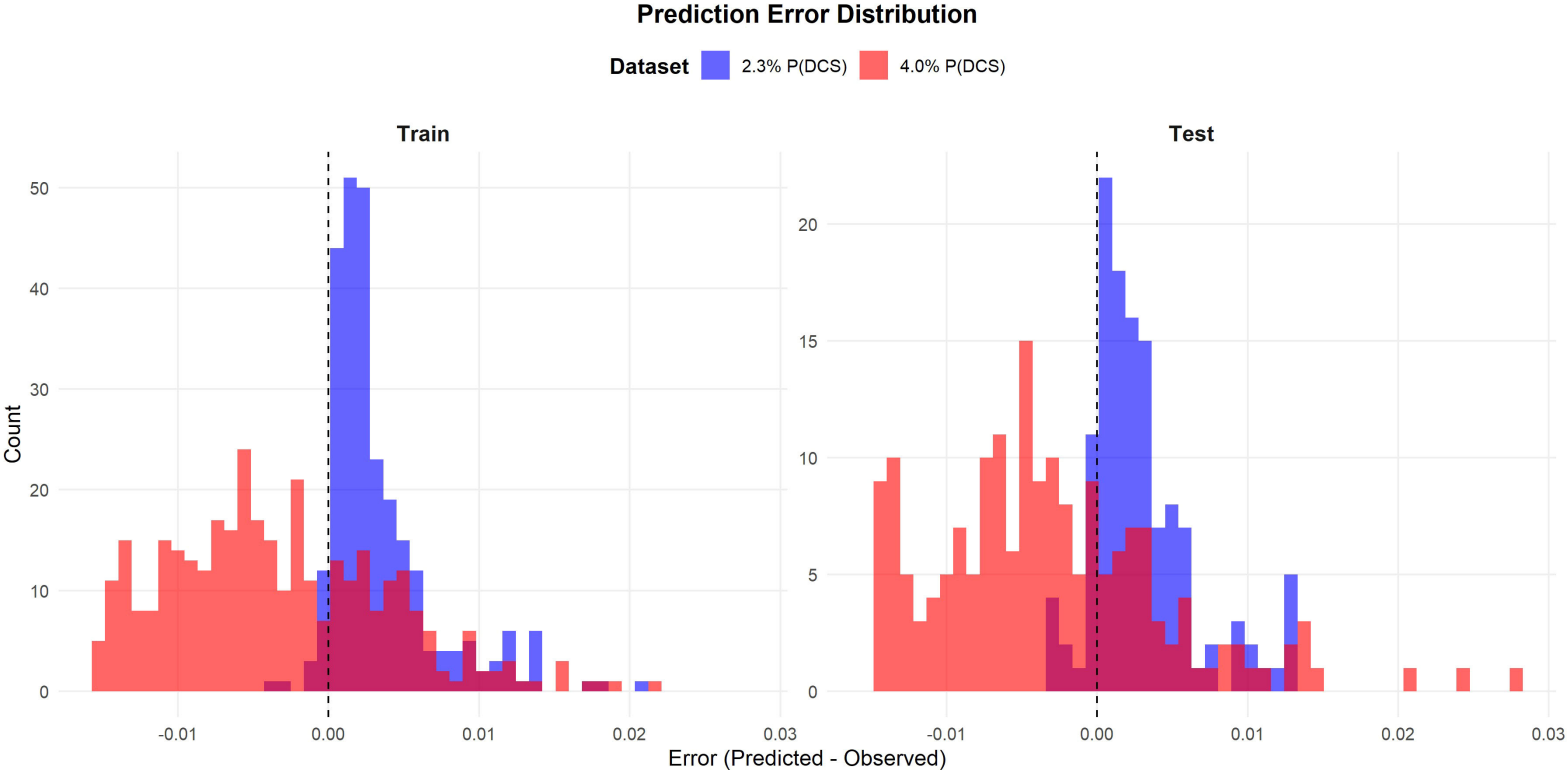
Error distribution for each expected P(DCS) group. The accuracy of the overall model is not the same for different expected P(DCS), probably due to the lower accuracy observed in the 100fsw (30msw) to 130fsw (39msw) range, which was more pronounced in the 4% P(DCS) dataset, as can be viewed in **Figure 5**. Additionally, shorter bottom times tend to produce slightly lower accuracy in the group with higher expected P(DCS), as demonstrated in **Figure 4**, where P(DCS) tends to be slightly overestimated. This performance difference can be observed when errors for different training sets are calculated separately (**Table 5**). It is clear that the lack of performance in the range above [100fsw (30msw) to 130fsw (39msw)] in the 4% P(DCS) training set affects the overall prediction accuracy for ${\hat{P}}_{j}$.

Interestingly, probably due to the nature of the optimization model, the error distribution is different for the training datasets, where ${\hat{P}}_{j}$ is slightly overestimated for P(DCS) 2.3% and underestimated for P(DCS) 4% (see **Figure 3**).

**Table 5:** Model accuracy.

	Full dataset	Training set	Test set
RMSE	0.694%	0.683%	0.702%
MAE	0.535%	0.528%	0.531%

**Figure 4:**
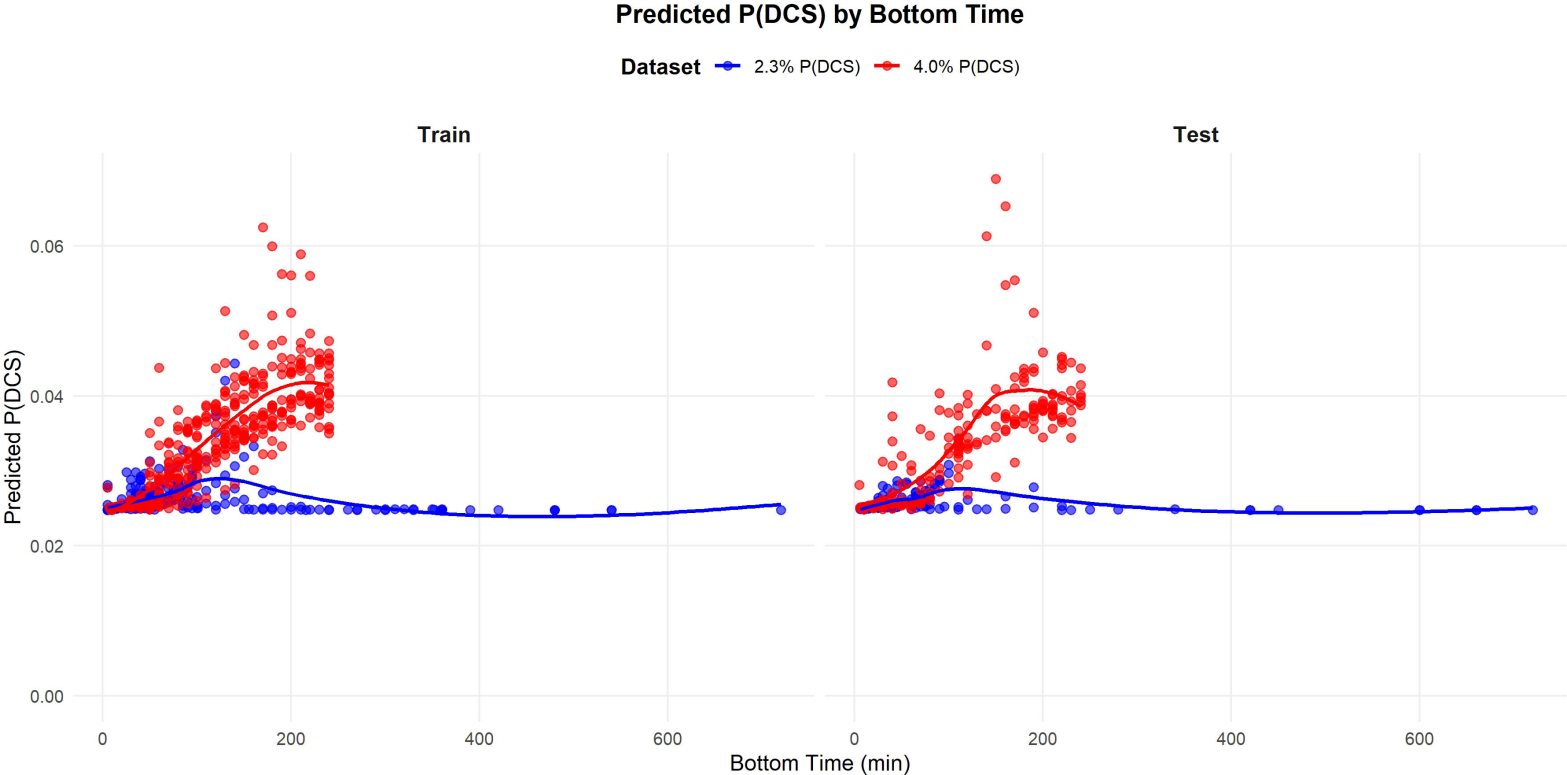
Error as a function of bottom time according to the overall length of the dive. Shorter bottom times tend to result in a slightly lower accuracy for the higher estimated P(DCS) group of profiles. Each colored line is a LOESS-smoothed trend curve of the absolute prediction error as a function of bottom time.

**Figure 5:**
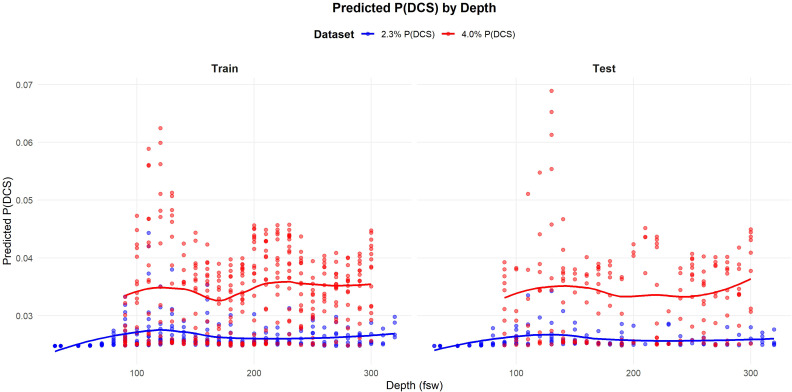
Error versus depth for each group of expected P(DCS) as calculated for the test set. As can be observed, the outliers are concentrated in the range of 100fsw to 130fsw, for both expected P(DCS). Each colored line is a LOESS-smoothed trend curve of the absolute prediction error as a function of bottom time.

Overall, except for the outliers highlighted in **Figures 2** and **3**, the overall adherence of the predicted values ${\hat{P}}_{j}$, supported by the low calculated mean error, results in a good predictivity of the estimated risk of decompression sickness when applied to the test set (**Figure 4**). As previously mentioned, the outliers shown in **Figure 4** are the same as those highlighted in **Figures 3** and **5** and are caused by the 100fsw (30msw) to 130fsw (39msw) dive profiles, for which the model is predicting a much higher P(DCS) than the ones estimated using the XVal-He-9 tables.

### 3.2 Out-of-sample results (reality check)

Finally, the trained model was applied to a set of dives for which the P(DCS) was known (**Table 6**). More than an accuracy check, which was made based on the train and test datasets and described above, the objective was simply to check for the overall adherence of the predicted values to reality. Additionally, compartment on-gassing and wash-out dynamics was plotted in order to observe when the gas kinetics changes between exponential and linear, according to Equations 1 and 2, so that the effect of the compartment crossover pressure *P_XO_* could be identified.

**Table 6:** Accuracy per training set.

	P(DCS) 2.3%	P(DCS) 4%
RMSE	0.491%	0.814%
MAE	0.348%	0.675%

**Figure 6:**
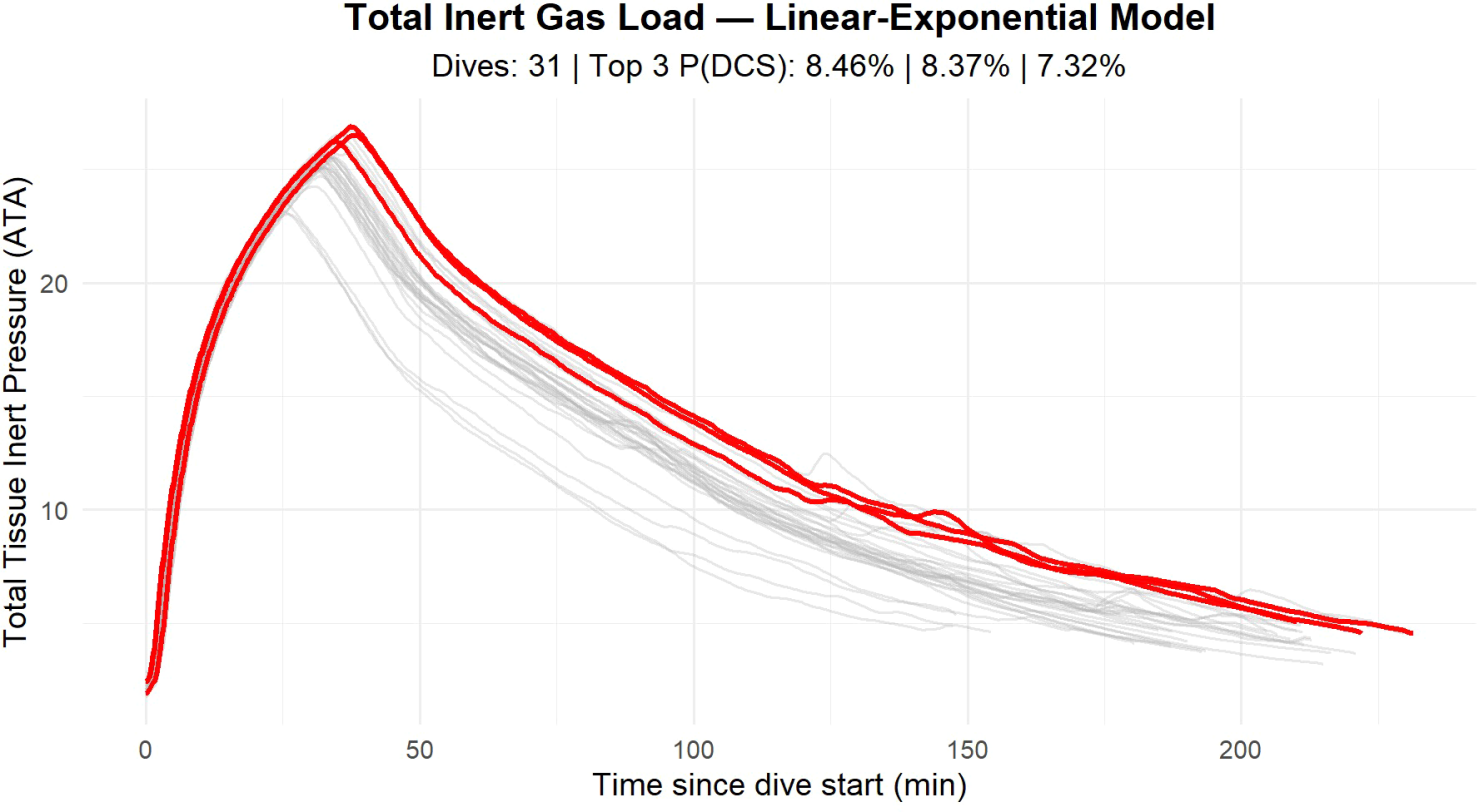
Sum of compartment inert gas pressures for all dive profiles used in the out-of-sample test. The red lines represent the dive profiles with higher expected P(DCS).

All profiles have significantly high expected P(DCS), as demonstrated in **Table 6**, with an estimated mean probability of 4.86%, reaching probabilities as high as 8% in some cases. **Figure 6** demonstrates the sum of all compartment pressures for all dives, highlighting the profiles with high estimated risk.

**Figure 7:**
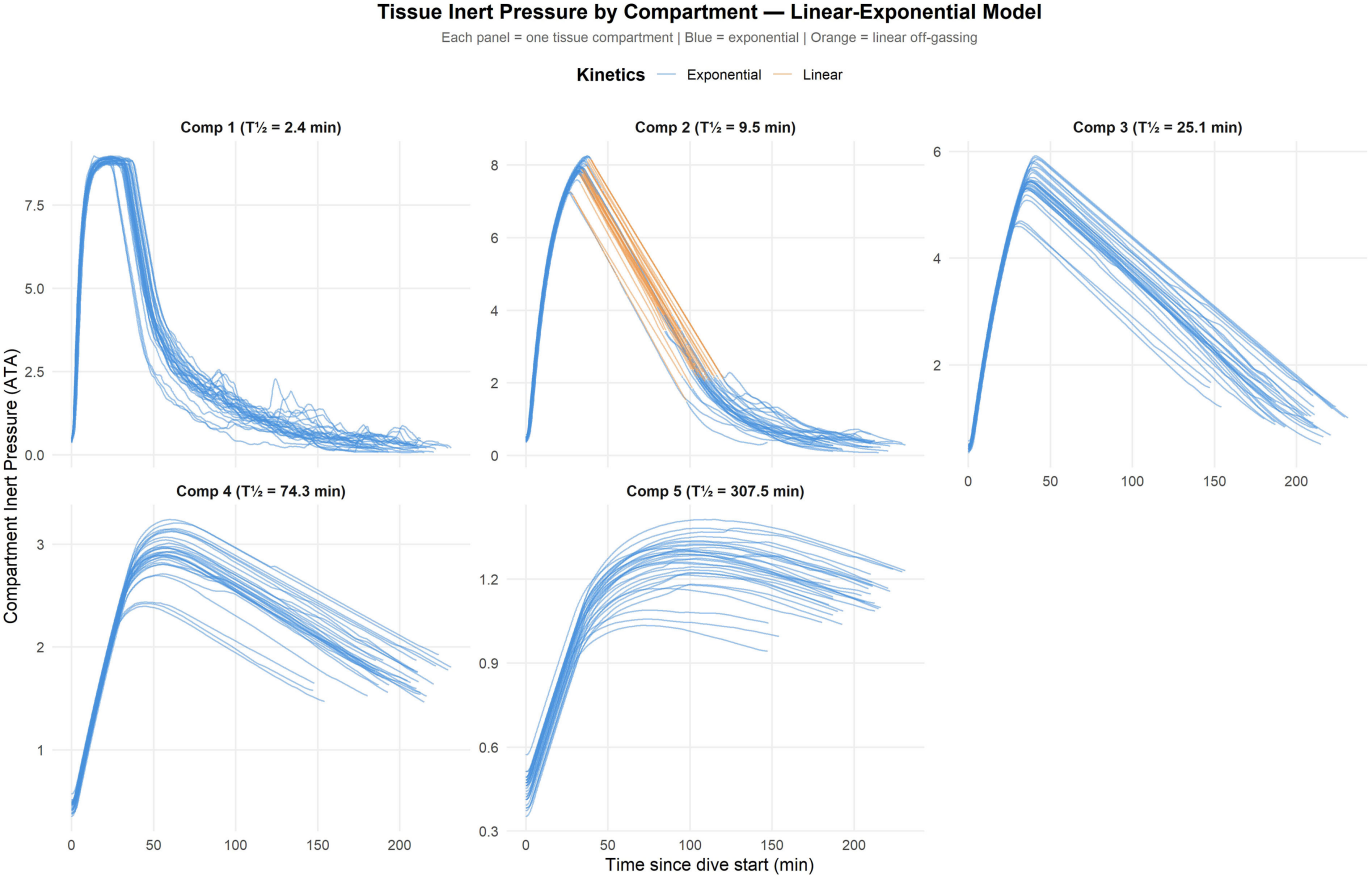
Total compartment pressure for the 31 dives analyzed. Orange lines are used to highlight the moments when the linear gas washout was triggered during decompression. Linear kinetics is only observed in compartment 2 since *P_XO_* was defined as zero for compartments 1, 3, 4, and 5.

The activation of the linear off-gassing dynamics, when the conditions described in Equations 1 and 2 were met, is shown in **Figure 7**, where the saturation and desaturation dynamics for each compartment are presented. Given that the crossover pressure *P_XO_* was considered to be zero for all but compartment number 2, this is thus the only compartment where a linear dynamics will be observed.

**Figure 8:**
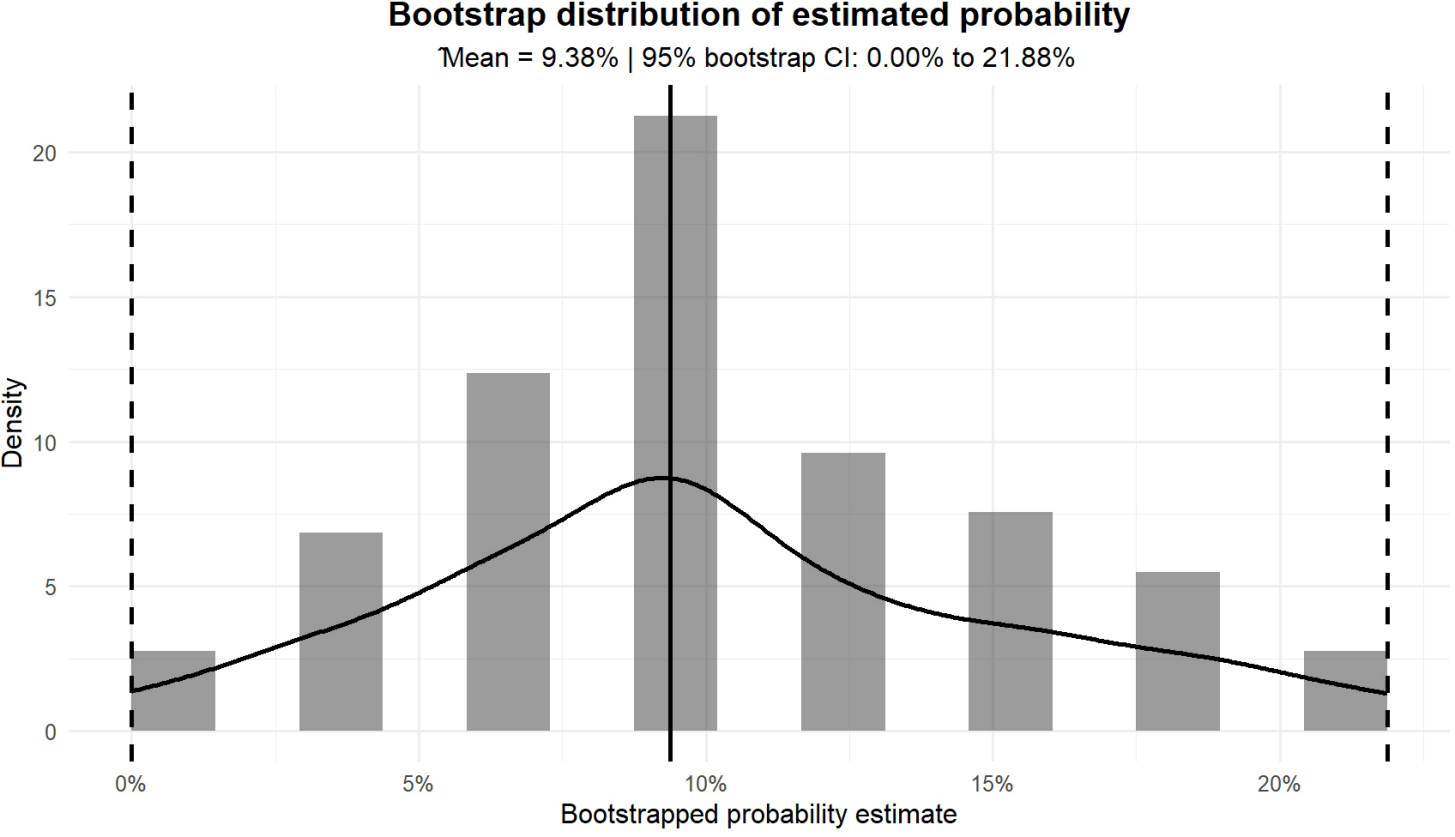
Bootstrap estimation of P(DCS) for the 31 profiles.

According to **Table 7**, these dive profiles provoked three cases of decompression sickness in a total of 31 dives, which leads to an observed P(DCS) of 9.3%. Given the small sample size, the actual probability can be anything between approximately zero to 19.5%, when the binomial standard error is accounted for. A bootstrap resampling simulation of 1,000 rounds produces a mean P(DCS) of 9.3% (**Figure 8**) and 95% confidence interval of [0, 18.8%]. Although the sample size is insufficient for parameter calibration, the elevated incidence of DCS events offers a valuable stress test of the model’s extrapolation capability since the training dataset contains substantially lower baseline probabilities (less than half of those observed here).

**Table 7:** Set of dives with high observed P(DCS) values.

	Mean	Max	Min	SE
Average depth (m)	91	91	90	0.09
Average time (min)	199	231	147	22
Estimated P(DCS)	4.86%	8.46%	2.62%	0.31%
Observed P(DCS)^a^	9.38%	19.47%	0.00%	5.15%

^a^For 95% confidence interval.

Another important observation drawn from these profiles is that the loss function, as designed in the present study, ignores any carryover effect from repetitive diving, which in this specific case seems to play an important role. **Figure 8** details compartment pressure and associated ${\hat{P}}_{j}\left(\text{DCS}\right)$ for the three dives that resulted in decompression sickness symptoms. It also reports the same information for the dives made in the previous 24 to 48 h by the same divers, which, interestingly, had higher estimated ${\hat{P}}_{j}$. Therefore, the high DCS incidence observed here might be related to the repetitive exposures rather than to a simple dive P(DCS).

## 4 Discussion

In the present study, the gradient-descent-based optimization model was based on pre-constructed XVal-He-9 tables. The utilization of gradient-descent-based optimization was possible in this study due to the methodology adopted, where, instead of using a dataset of dives and outcomes for training, the model was based on pre-constructed XVal-He-9 tables. The adoption of tables with known (or estimated, which, for the purposes of this study, will be assumed to be equal) values allowed the problems of non-smoothness demonstrated by Howle et al. to overcome (Howle et al., 2009), which was related to the binary and low-incidence nature of DCS, causing the gradient-descent-based algorithms to run into numerical problems and, consequently, obtain low accuracy. Using profiles and their related P(DCS), instead of dives and observed cases, is equivalent to analyzing a much greater number of dives (for 3% incidence, 924 profiles would be equivalent to approximately 35,000 real dives), addressing the low incidence issue and substantially improving the accuracy of the model. Of course, it must be taken into account that the XVal-He-9 tables are themselves the result of an optimization process, and as such, actual P(DCS), observed if the profiles were sufficiently tested, might differ than the P(DCS) calculated using the XVal-He-9 algorithm. In reality, this potential diversion might be the cause for the low accuracy observed in the 100- to 130-ft-depth profiles, especially for bottom times between 150 and 200 min, a region where the ${\hat{P}}_{j}\left(\text{DCS}\right)$ is almost twice the P(DCS) used for training.

**Figure 9:**
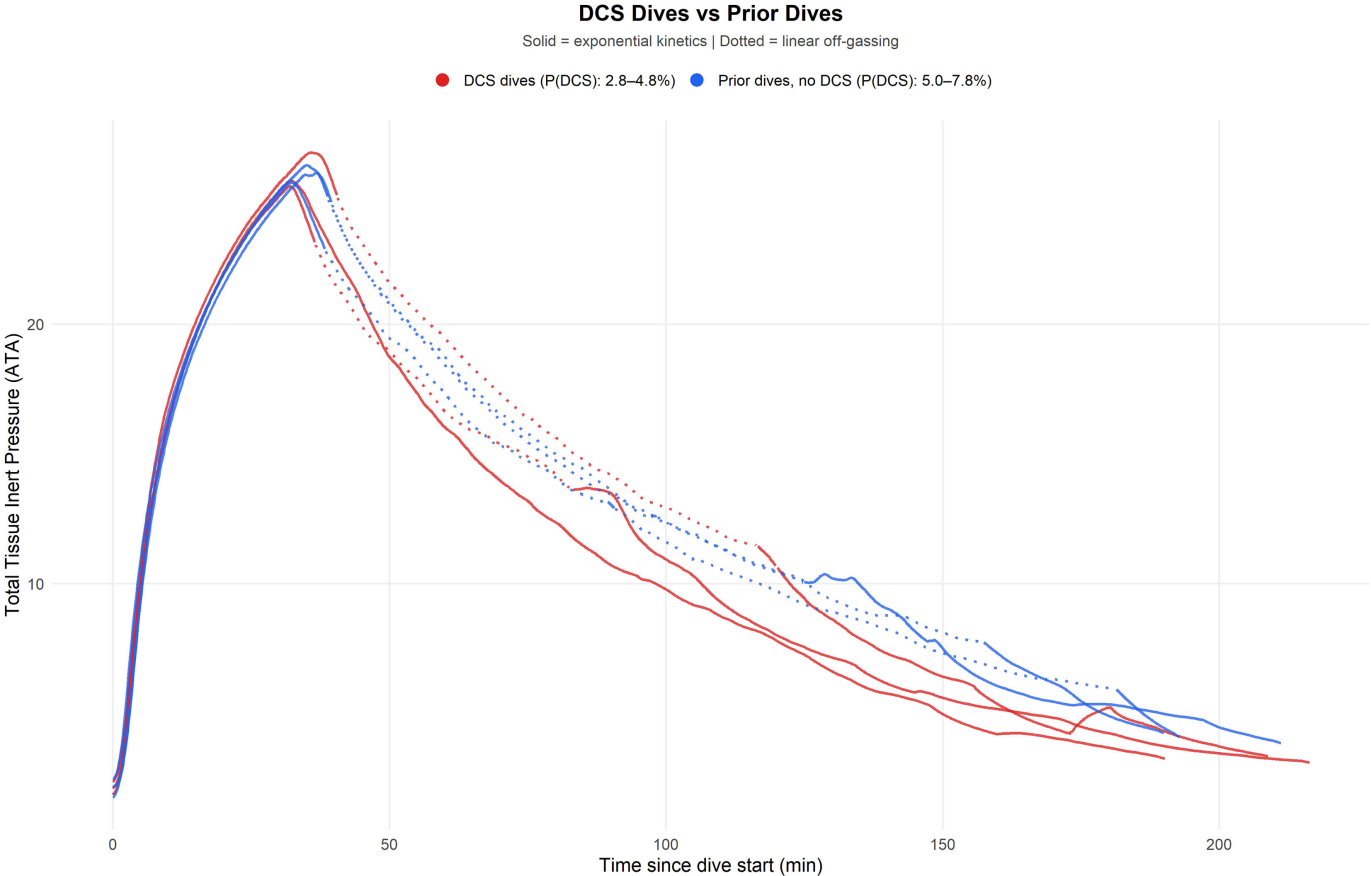
Dives that resulted in DCS and dives made in the previous 24- to 48-h intervals by the same divers.

One interesting feature of the present method is that the loss function (Equation 14) can be further adjusted in order to account for variables other than pure tissue saturation and gas kinetics. The repetitive dive effect observed in **Figure 9** is very illustrative. From the simple gas saturation standpoint, surface intervals equal or longer than 24 h cause the compartment pressures to be neglectable. However, a recent study (Balestra et al., 2024) demonstrated the carryover effect of multiple days of deep-sea diving, observing cumulative increases in pro-inflammatory markers (neopterin and interleukin-6), with uncertain implications to increased risk of decompression sickness, while another study demonstrated that even similar exposures, with slightly different decompression profiles, can produce different physiological responses (Schirato et al., 2024). It might be speculated that such carryover effect is behind the DCS cases observed in the out-of-sample application.

Other individual or dive-related indicators can potentially be added to the loss function, such as age, gender, cold-induced perfusion reduction, etc. On the one hand, the addition of extra factors could lead to a more individualized risk function. However, on the other hand, these might lead to a process that is harder to converge and that would require stronger procedures and probably demand a larger training dataset.

Unlike previous models based on three compartments, the current study employs a five-compartment structure whose half-times were derived from physiological tissue data rather than optimized during fitting. This formulation results in 12 optimized parameters, exceeding the number used in earlier publications. However, the larger parameter space is justified by the correspondingly large size of the observational dataset.

## 5 Conclusion and limitations

In conclusion, gradient-based optimization, trained based on existing probabilistic tables, seems to be capable of satisfactorily predicting decompression sickness risk for a given dive profile; however, additional testing would be needed to corroborate the accuracy levels calculated in this study. Of course, as in any probabilistic approach, the method described in the present study ignores other potential factors known to affect the outcome of a given exposure, such as thermal comfort, exercise level, and hydration, among others. Therefore, it should be taken as a framework capable of estimating decompression sickness occurrence rather than a precise and definitive predictor for decompression outcome on an individual basis.

## Data Availability

The original contributions presented in the study are included in the article/supplementary material. Further inquiries can be directed to the corresponding author.
